# A Case of Parasitic Leiomyoma After Laparoscopic Myomectomy With Power Morcellator

**DOI:** 10.7759/cureus.77519

**Published:** 2025-01-15

**Authors:** Yoko Aoyagi, Kaei Nasu, Tomoko Hirakawa, Saki Aso, Eiji Kobayashi

**Affiliations:** 1 Department of Obstetrics and Gynecology, Oita University, Yufu, JPN

**Keywords:** follow-up, laparoscopic myomectomy, late complication, parasitic leiomyoma, power morcellation

## Abstract

As the growth of morcellated leiomyoma fragments is slow, iatrogenic parasitic leiomyomas are recognized as late complications of laparoscopic myomectomies. We present a case of parasitic leiomyoma diagnosed several years following laparoscopic myomectomy using a power morcellator. A 48-year-old Japanese woman presented with a pelvic mass. At 36 years old, she underwent laparotomic myomectomy using a power morcellator for uterine leiomyomas. Twelve years after the initial laparoscopic myomectomy, multiple subserous leiomyomas were detected. She was treated by open surgery. A solid tumor was found in the pouch of Douglas, which was firmly adhered to surrounding tissues. It was diagnosed as a parasitic leiomyoma. Gynecologists should be aware of the risk of iatrogenic parasitic leiomyoma development as a late complication of laparoscopic myomectomy using a power morcellator. Moreover, patients should be followed up at least until menopause.

## Introduction

Uterine leiomyoma is the most common benign gynecological tumor, affecting as many as 20-50% of women of reproductive age. It is also present in approximately 80% of all hysterectomy specimens [1,2]. These benign neoplasms are composed of smooth muscle cells and fibrous connective tissues.

In rare cases, uterine leiomyoma can lose the connection with the uterus and instead attach to intra-abdominal or pelvic tissues such as the pelvic peritoneum, abdominal wall, small intestine, colon, and omentum [3-7]. In such cases, they are known as parasitic leiomyomas and are classified into spontaneous/primary and iatrogenic/secondary forms. One rare subtype of pedunculated subserosal leiomyoma can form from a primary parasitic leiomyoma and develop into a large stalk. They adhere to surrounding organs, such as the broad ligament or omentum, and develop their own collateral blood supply. Thus, the leiomyomas can grow after detaching from the uterus [3-5]. An alternative mechanism for the development of primary parasitic leiomyomas is associated with the metaplasia of the peritoneum [3].

The prevalence of laparoscopic myomectomy and hysterectomy has led to the discovery of a new type of parasitic leiomyoma: iatrogenic parasitic leiomyoma [4]. These laparoscopic approaches have several advantages over conventional open approaches, such as decreased blood loss, less postoperative pain, fewer postoperative complications, faster recovery time, and shorter hospital stays [5,8,9]. Power morcellators are often used to fragment bulky uterine leiomyomas that would otherwise not be removed through a smaller incision. The process involves dividing the leiomyomatous tissue using a rotating circular blade to facilitate the removal of the specimen. Even with correct use and careful observation, some minute particles may accidentally be left behind where they can settle and implant in the peritoneal cavity [4,5,7,10]. They then receive a blood supply from nearby organs and develop into parasitic leiomyomas [4]. Most of the iatrogenic parasitic leiomyomas reported have been located in the pelvis, suggesting that gravity may be responsible for the movement of leiomyoma fragments to the pelvic cavity. To avoid the development of iatrogenic parasitic leiomyoma, in-bag morcellation is proposed [11].

Owing to the increase in laparoscopic myomectomies using power morcellators, several cases of parasitic leiomyomas related to this technique have been reported in the literature. Herein, we report a case of iatrogenic pelvic parasitic leiomyoma diagnosed 12 years after laparoscopic myomectomy using a power morcellator.

## Case presentation

A 48-year-old Japanese woman, gravida 1, para 1, was referred to our hospital for a thorough examination of a pelvic mass. At 36 years old, she underwent laparotomic myomectomy using a power morcellator for uterine leiomyomas. At 40 years old, she gave birth to a healthy daughter via cesarean section. Thereafter, gynecological examinations were not performed.

**Figure 1:**
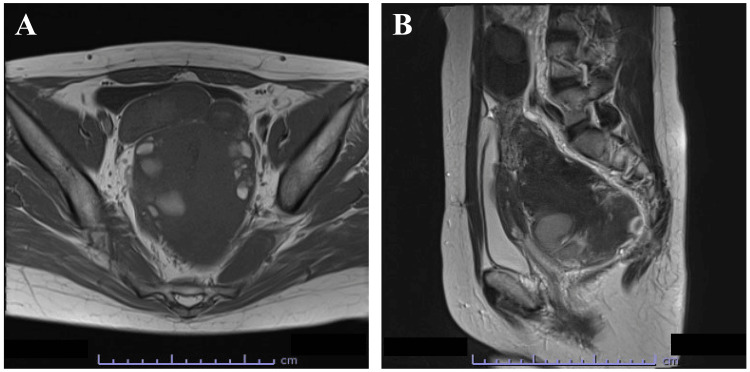
T1-weighted magnetic resonance imaging findings in the pelvic tumor. (A) Horizontal view. (B) Sagittal view. The size of the largest leiomyoma nodule in the pouch of Douglas was 14.5 x 9.5 x 9.4 cm. The hemorrhagic cysts inside the tumor were located in the pouch of Douglas, and the continuity with the uterus was unclear. Both ovaries and fallopian tubes were normal. Ascites was not observed.

Twelve years after the initial laparoscopic myomectomy, multiple subserous leiomyomas were detected using transvaginal ultrasonography and magnetic resonance imaging (Figure 1). The largest leiomyoma nodule (14.5 x 9.5 x 9.4 cm) that contained hemorrhagic cysts inside the tumor was detected in the pouch of Douglas. The continuity between the largest leiomyoma nodule and the uterus was unclear. Blood sampling revealed a cancer antigen (CA) 125 concentration of 124.5 U/mL and a CA19-9 concentration of 295.8 U/mL.

**Figure 2:**
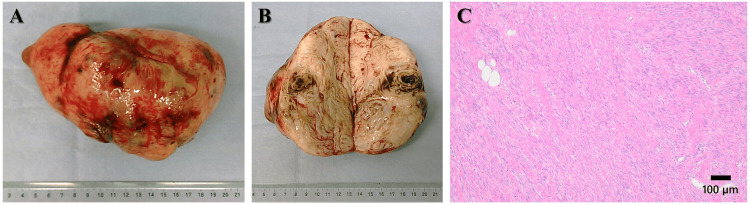
Gross and microscopic findings of the pelvic tumor. (A) The resected tumor in the pouch of Douglas was solid. (B) The cut surface of the resected tumor revealed hemorrhagic cysts inside the tumor. (C) The resected tumor consists of benign smooth muscle cells separated by a dense fibrovascular stroma (hematoxylin and eosin staining).

The patient was treated by open surgery. A solid tumor had developed in the pouch of Douglas, which adhered firmly to the surrounding organs (Figure 2A, 2B). There was no continuity between the solid tumor and the uterus. A few subserous and intramural leiomyomas were observed in the uterine corpus. Both ovaries and fallopian tubes were normal. Histologically, the resected tumor in the pouch of Douglas was well circumscribed, with the typical features of a benign leiomyoma (Figure 2C).

The patient’s postsurgical course was unremarkable. Six years after surgery, there was no evidence of recurrence. The patient gave her written informed consent to present this case.

## Discussion

The reported incidence of iatrogenic parasitic leiomyomas after laparoscopic morcellation is 0.07-1.25% [5,6,8-10]. However, the prevalence of spontaneous parasitic leiomyomas has been estimated at 0.21% [8]. Iatrogenic parasitic leiomyomas are now considered a late complication of laparoscopic myomectomy with an average diagnostic interval of 4-5.7 years [5,7-9]. There are often multiple parasitic leiomyoma lesions of varying sizes (range, 0.8-30 cm). Tan-Kim et al. [6] reported that an age <40 years is associated with a higher risk of developing parasitic leiomyomas after power morcellation. These findings are consistent with the hypothesis that small leiomyoma fragments grow slowly into a large size.

Parasitic leiomyomas usually remain asymptomatic until pressure symptoms develop [5]. Most small parasitic leiomyomas are found incidentally during other investigations or surgical procedures [10]. In contrast, three-quarters of patients with parasitic leiomyoma present with symptoms such as abdominal pressure, abdominal distension, abdominal or pelvic pain, constipation, urinary frequency, and dyspareunia [4,5,7,10].

Surgical removal is recommended for symptomatic or large parasitic leiomyomas, either through open surgery or laparoscopy [7]. Although the prognosis is very good, with a low recurrence rate, reoperation for diagnosis and treatment is associated with significant morbidity. Debulking procedures, such as omentectomy, appendectomy, or bowel resection, are necessary to eliminate all parasitic leiomyomas in 10% of patients [7,9].

As uterine leiomyomas are estrogen-dependent, slow-growing tumors, iatrogenic parasitic leiomyomas may gradually regress after menopause due to the lack of ovarian hormones. Moreover, parasitic leiomyomas are commonly reported in premenopausal women. We present a case of iatrogenic parasitic leiomyoma diagnosed long-term after laparoscopic myomectomy using power morcellator. Gynecological examinations were not performed after giving birth.

## Conclusions

Gynecologists should be aware of the risk of iatrogenic parasitic leiomyoma development as a late complication of laparoscopic myomectomy with power morcellator, especially without in-bag morcellation. To avoid such complications, in-bag morcellation is proposed. Moreover, patients should be followed up at least until menopause.
